# Serum uric acid was non-linearly associated with the risk of all-cause and cardiovascular death in individuals with coronary heart disease: a large prospective cohort study

**DOI:** 10.3389/fendo.2023.1278595

**Published:** 2023-12-13

**Authors:** Xuejiao Yan, Jing Gong, Zhenwei Wang, Qiyong Wu, Chunjian Qi, Fangfang Wang

**Affiliations:** ^1^ Department of Cardiology, The Affiliated Changzhou No.2 People’s Hospital of Nanjing Medical University, Changzhou, Jiangsu, China; ^2^ Department of Geriatrics, Nanjing Tongren Hospital, School of Medicine, Southeast University, Nanjing, China; ^3^ Department of Cardiology, The First Affiliated Hospital of Zhengzhou University, Zhengzhou, China; ^4^ Department of Thoracic and Cardiac Surgery, The Affiliated Changzhou No.2 People’s Hospital of Nanjing Medical University, Changzhou, Jiangsu, China; ^5^ Medical Research Center, The Affiliated Changzhou No.2 People’s Hospital of Nanjing Medical University, Changzhou, Jiangsu, China

**Keywords:** uric acid, coronary heart disease, all-cause death, cardiovascular death, mortality

## Abstract

**Objective:**

To investigate the association of serum uric acid (SUA) with all-cause and cardiovascular death in individuals with coronary heart disease (CHD).

**Methods:**

In this prospective cohort study, 1556 individuals from the National Health and Nutrition Examination Survey (1999-2015) were included in the analysis. Multivariate COX regression analysis, restricted cubic spline plot (RCS) and threshold effect were used to investigate the association between SUA and all-cause and cardiovascular death in individuals with CHD.

**Results:**

In the fully adjusted model, when SUA was regarded as a continuous variable, it was closely associated with the risk of all-cause and cardiovascular death (P < 0.01). When all participants were divided into four groups according to the quartile of SUA, compared with Q1 group, only individuals in Q4 group had higher risk of all-cause and cardiovascular death (P = 0.002 and 0.034). The following subgroup analysis showed that the association between SUA and all-cause death risk was still statistically significant in individuals over 60 years old, male, with hypertension, without diabetes and with chronic kidney disease, while the association with cardiovascular death risk only persisted in individuals over 60 years old and male (P < 0.05). Further sensitivity analysis showed that SUA was still closely associated with all-cause and cardiovascular death, whether as a continuous variable or a classified variable (P = 0.007 and 0.044). RCS analysis revealed that SUA had a nonlinear association with all-cause and cardiovascular death risk (P for nonlinearity < 0.01). Threshold effect analysis showed that SUA below 345 umol/L was negatively associated with all-cause and cardiovascular death risk (P < 0.05), while SUA above 345 umol/L was positively associated with all-cause and cardiovascular death risk (P < 0.001), and the 2-piecewise regression model was better than the 1-line regression model (P for likelihood ratio test < 0.05).

**Conclusion:**

SUA had a nonlinear association with all-cause and cardiovascular death risk in individuals with CHD.

## 1 Introduction

Serum uric acid (SUA), as an end product of purine metabolism mainly excreted by kidney, has been recognized as a risk factor for gout, gouty arthritis and kidney calculi (1–4). In addition, the current research background has also confirmed that SUA is closely related to the prevalence and incidence of metabolic syndrome, cardiovascular disease, kidney disease and cancer (5–7). Of course, these diseases mainly occur in patients with high levels of SUA. However, its correlation with mortality has not been agreed, among which all-cause death and cardiovascular death are the most concerned. Some studies show that higher levels of SUA are related to higher risk of all-cause death or cardiovascular death (8, 9), while others suggest that lower levels of SUA are related to higher risk of all-cause death or cardiovascular death, and some studies have even found that SUA levels are not associated with the risk of all-cause or cardiovascular death (10–12). Nevertheless, most studies tend to show an approximately U-shaped correlation between SUA and the risk of all-cause death or cardiovascular death.

However, the current research mainly focuses on the relationship between SUA and the death risk of the general population or healthy population, and does not explore the population with coronary heart disease (CHD) too much. Therefore, this study aimed at exploring the correlation between SUA and all-cause death and cardiovascular death for individuals with CHD from the National Health and Nutrition Examination Survey (NHANES).

## 2 Materials and methods

### 2.1 Study population

**Figure 1:**
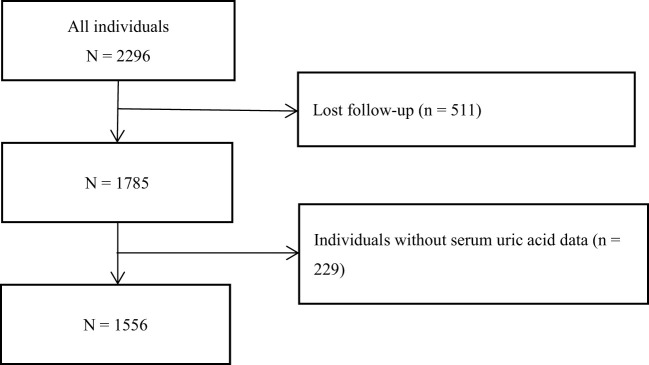
Flow chart of selected patients.

All participants in this study came from the NHANES from 1999 to 2015. As shown in **Figure 1**, after excluding the individuals who lost their visits and did not have SUA data, 1556 individuals were finally included in this study for the following statistical analysis. The research scheme was approved by the National Center for Health Statistics of the Center for Disease Control and Prevention Institutional Review Board. Participants have signed the informed consent form. The research program and content were in line with the Declaration of Helsinki.

### 2.2 Data collection and definitions

In this prospective cohort study, we collected some baseline data according to the design of the research scheme, such as demographic data, physical measurement data, complications and drug treatment data and biomarker data. In this study, smoking was defined as a binary variable according to the current smoking situation: yes or no. The definition of drug treatment data was the same as above. Hypertension was defined as hypertension diagnosed by doctors or current systolic blood pressure (SBP)/diastolic blood pressure (DBP) ≥ 140/90 mmHg or taking antihypertensive drugs. Diabetes was defined as diabetes diagnosed by doctors or current fasting plasma glucose (FPG) ≥ 7.0 mmol/L or hemoglobin A1c (HbA1c) ≥ 6.5% or being treated with hypoglycemic drugs. Hypercholesterolemia was defined as a medical history diagnosed by a doctor or currently being treated with cholesterol-lowering drugs. Stroke was defined as a self-reported history of stroke. Chronic kidney disease (CKD) was defined as estimated glomerular filtration rate (eGFR) < 60 ml/min/1.73m^2^, in which eGFR was estimated according to published literature (13).

All participants were followed up from the date of NHANES interview to December 31, 2015, and the outcomes included all-cause death and cardiovascular death defined by ICD-10 codes.

### 2.3 Statistical analysis

In this study, we divided all participants into four groups according to the quartile of SUA: Q1 ≤ 297.4 umol/L, 297.4 umol/L< Q2 ≤ 356.9 umol/L, 356.9 umol/L< Q3 ≤ 422.3 umol/L, Q4 ≥ 422.3 umol/L. One-way ANOVA or Kruskal-Wallis H analysis were used to test the differences of each continuous variable between groups. Chi-square test was used to analyze the differences of each classified variable between groups. Kaplan-Meier survival analysis was used to test the cumulative incidence of all-cause death and cardiovascular death among the four groups of SUA. COX regression analysis was used to test the correlation between SUA and all-cause death and cardiovascular death. Subgroup analysis and sensitivity analysis were used to test the stability of the correlation between SUA and all-cause death and cardiovascular death. Restricted cubic spline plot (RCS) and threshold effect analysis were used to test the potential nonlinear correlation, threshold effect and saturation effect between SUA and all-cause death and cardiovascular death. The above statistical tests were conducted by SPSS 26.0 or R 4.1.3, and a two-tailed P value < 0.05 was considered statistically significant.

## 3 Results

### 3.1 Baseline characteristics

**Figure 2:**
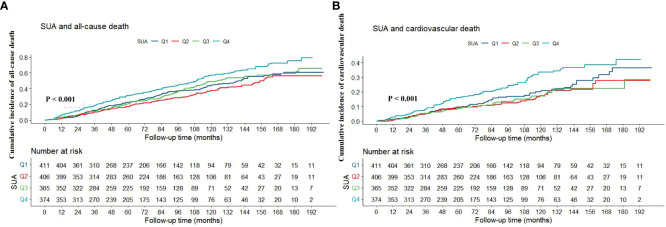
Kaplan-Meier survival curve of association between SUA with all-cause **(A)** and cardiovascular death **(B)**. SUA, serum uric acid.

As shown in **Table 1**, the levels or probabilities of variables such as age, sex, smoking, hypertension, diabetes, hypotensive drugs, hypoglycemic drugs, BMI, TG, HDL-C, HB, PLT, ALT, TBIL, eGFR, CRP, HbA1c, all-cause death and cardiovascular death were significantly different among the four groups of SUA. And the proportion and cumulative incidence of all-cause and cardiovascular death in Q4 group were higher than that in Q1 group (P < 0.05) (**Table 1**; **Figure 2**).

**Table 1:** Baseline characteristics by the quartile of the SUA.

	Total population	Q1	Q2	Q3	Q4	P value
Age, years	68.66 ± 11.56	67.27 ± 12.92	68.90 ± 10.54	68.95 ± 11.67	69.63 ± 10.83	0.030
Sex, male, n (%)	1048 (67.40)	215 (52.30)	274 (67.50)	282 (77.30)	277 (74.10)	< 0.001
Smoking, n (%)						0.001
Yes	268 (26.70)	84 (34.00)	80 (30.10)	58 (23.80)	46 (18.70)	
No	735 (73.30)	163 (66.00)	186 (69.90)	186 (76.20)	200 (81.30)	
Comorbidities, n (%)						
Hypertension						< 0.001
Yes	1125 (72.60)	265 (65.00)	283 (70.00)	271 (74.20)	306 (82.00)	
No	425 (27.40)	143 (35.00)	121 (30.00)	94 (25.80)	67 (18.00)	
Diabetes						0.010
Yes	491 (31.60)	134 (32.70)	124 (30.50)	94 (25.80)	139 (37.20)	
No	1063 (68.40)	276 (67.30)	282 (69.50)	270 (74.20)	235 (62.80)	
Hypercholesterolemia						0.214
Yes	1046 (70.90)	267 (69.70)	286 (73.10)	254 (73.40)	239 (67.30)	
No	2987 (65.30)	753 (67.80)	740 (64.40)	777 (67.70)	717 (61.40)	
Stroke						0.392
Yes	238 (15.30)	73 (17.80)	55 (13.60)	53 (14.60)	57 (15.20)	
No	1316 (84.70)	338 (82.20)	350 (86.40)	311 (85.40)	317 (84.80)	
Treatment, n (%)						
Hypotensive drugs						< 0.001
Yes	1025 (68.30)	241 (60.70)	262 (66.50)	243 (69.40)	279 (77.50)	
No	476 (31.70)	156 (39.30)	132 (33.50)	107 (30.60)	81 (22.50)	
Hypoglycemic drugs						0.017
Yes	458 (29.50)	126 (30.70)	115 (28.30)	88 (24.10)	129 (34.50)	
No	1097 (70.50)	284 (69.30)	291 (71.70)	277 (75.90)	245 (65.50)	
Cholesterol-lowering drugs						0.108
Yes	916 (65.70)	225 (63.70)	262 (68.90)	223 (68.40)	206 (61.50)	
No	478 (34.30)	128 (36.30)	118 (31.10)	103 (31.60)	129 (38.50)	
BMI, kg/m^2^	29.41 ± 6.14	27.93 ± 5.80	28.71 ± 5.76	30.16 ± 6.02	31.05 ± 6.51	< 0.001
SBP, mmHg	132.69 ± 21.87	133.52 ± 23.51	133.55 ± 21.62	132.04 ± 20.24	131.46 ± 21.80	0.469
DBP, mmHg	66.97 ± 12.62	66.91 ± 12.74	66.84 ± 11.66	67.49 ± 13.10	66.68 ± 13.05	0.849
TG, mmol/L	1.46 (1.03, 2.15)	1.37 (0.97, 1.94)	1.45 (1.00, 2.12)	1.67 (1.13, 2.27)	1.68 (1.20, 2.81)	< 0.001
TC, mmol/L	4.69 ± 1.22	4.73 ± 1.16	4.66 ± 1.22	4.67 ± 1.18	4.70 ± 1.32	0.865
LDL−C, mmol/L	2.58 ± 0.96	2.58 ± 0.98	2.53 ± 0.95	2.65 ± 1.00	2.55 ± 0.89	0.664
HDL−C, mmol/L	1.24 ± 0.37	1.34 ± 0.40	1.24 ± 0.37	1.19 ± 0.31	1.17 ± 0.36	< 0.001
WBC, *10^9^/L	7.42 ± 2.57	7.39 ± 2.22	7.37 ± 2.62	7.25 ± 2.63	7.70 ± 2.78	0.100
HB, g/dL	14.03 ± 1.60	13.84 ± 1.46	14.18 ± 1.57	14.21 ± 1.49	13.90 ± 1.82	0.001
PLT, *10^9^/L	227.91 ± 68.96	237.66 ± 73.08	228.11 ± 66.85	222.46 ± 68.56	222.29 ± 65.95	0.005
ALT, U/L	21.00 (16.00, 27.00)	18.00 (15.00, 24.75)	21.00 (17.00, 29.00)	22.00 (16.00, 28.00)	23.00 (17.00, 27.25)	0.015
AST, U/L	24.00 (20.00, 28.00)	23.00 (19.25, 26.00)	24.00 (21.00, 29.00)	23.00 (20.00, 29.00)	24.00 (21.00, 31.00)	0.069
TBIL, umol/L	12.74 ± 5.40	11.50 ± 4.82	12.77 ± 5.60	13.15 ± 5.41	13.67 ± 5.55	< 0.001
ALB, g/L	41.72 ± 3.36	41.66 ± 3.27	41.61 ± 3.60	42.01 ± 3.01	41.60 ± 3.52	0.287
eGFR, ml/min/1.73m^2^	77.79 ± 29.64	91.11 ± 33.29	80.73 ± 26.27	75.09 ± 24.56	62.61 ± 25.75	< 0.001
FIB, g/L	4.03 ± 0.85	3.98 ± 0.88	3.97 ± 0.92	3.98 ± 0.73	4.17 ± 0.86	0.318
CRP, mg/L	0.26 (0.12, 0.61)	0.20 (0.08, 0.53)	0.24 (0.10, 0.54)	0.25 (0.10, 0.58)	0.31 (0.15, 0.71)	0.009
FPG, mmol/L	6.85 ± 2.62	7.11 ± 3.20	6.81 ± 2.51	6.54 ± 2.23	6.93 ± 2.49	0.221
HbA1c, %	6.20 ± 1.26	6.30 ± 1.41	6.17 ± 1.26	6.05 ± 1.04	6.27 ± 1.26	0.028
Outcomes, n (%)						
All-cause death						< 0.001
Yes	604 (38.80)	151 (36.70)	130 (32.00)	137 (37.50)	186 (49.70)	
No	952 (61.20)	260 (63.30)	276 (68.00)	228 (62.50)	188 (50.30)	
Cardiovascular death						0.001
Yes	244 (15.70)	63 (15.30)	54 (13.30)	45 (12.30)	82 (21.90)	
No	1312 (84.30)	348 (84.70)	352 (86.70)	320 (87.70)	292 (78.10)	

Data were expressed as mean ± SD, median (first quartile, third quartile), or n (%). SUA, serum uric acid; BMI, body mass index; SBP, systolic blood pressure; DBP, diastolic blood pressure; TG, triglycerides; TC, total cholesterol; LDL-C, low-density lipoprotein cholesterol; HDL-C, high-density lipoprotein cholesterol; WBC, white blood cell; HB, hemoglobin; PLT, platelets; ALT, alanine transaminase; AST, aspartate aminotransferase; TBIL, total bilirubin; ALB, albumin; eGFR, estimated glomerular filtration rate; FIB, fibrinogen; CRP, C-reactive protein; FPG, fasting plasma glucose; HbA1c, hemoglobin A1c.

### 3.2 Association of SUA with all-cause and cardiovascular death

During the median follow-up period of 73 months, a total of 604 people died, of which 244 died of cardiovascular causes. As shown in **Table 2**. In the fully adjusted model (Model 3), when SUA was regarded as a continuous variable, it was closely associated with the risk of all-cause and cardiovascular death (HR: 1.002, 95% CI: 1.001-1.003, P < 0.001; HR: 1.002, 95% CI: 1.001-1.003, P = 0.002). When all participants were divided into four groups according to the quartile of SUA, compared with Q1 group, only individuals in Q4 group had higher risk of all-cause and cardiovascular death (HR: 1.433, 95% CI: 1.145-1.794, P = 0.002; HR: 1.430, 95% CI: 1.028-1.990, P = 0.034).

**Table 2:** Multivariate Cox regression analysis of SUA with all-cause and cardiovascular death.

	Model 1	P value	Model 2	P value	Model 3	P value
	HR (95% CI)		HR (95% CI)		HR (95% CI)	
All-cause death						
Q1	Ref.	–	Ref.	–	Ref.	–
Q2	0.816 (0.645, 1.032)	0.089	0.762 (0.602, 0.963)	0.023	0.837 (0.658, 1.064)	0.147
Q3	1.035 (0.821, 1.305)	0.768	0.935 (0.742, 1.178)	0.568	1.091 (0.860, 1.386)	0.472
Q4	1.441 (1.162, 1.786)	0.001	1.354 (1.092, 1.678)	0.006	1.433 (1.145, 1.794)	0.002
P for trend	–	< 0.001	–	< 0.001	–	< 0.001
per 1 unit increment	1.002 (1.001, 1.003)	< 0.001	1.002 (1.001, 1.003)	< 0.001	1.002 (1.001, 1.003)	< 0.001
Cardiovascular death						
Q1	Ref.	–	Ref.	–	Ref.	–
Q2	0.815 (0.566, 1.172)	0.270	0.764 (0.531, 1.100)	0.147	0.717 (0.497, 1.035)	0.076
Q3	0.814 (0.555, 1.193)	0.291	0.735 (0.501, 1.078)	0.115	0.776 (0.528, 1.139)	0.195
Q4	1.519 (1.094, 2.110)	0.013	1.427 (1.028, 1.982)	0.034	1.430 (1.028, 1.990)	0.034
P for trend	–	0.001	–	< 0.001	–	< 0.001
per 1 unit increment	1.002 (1.001, 1.003)	< 0.001	1.002 (1.001, 1.003)	0.002	1.002 (1.001, 1.003)	0.002

Model 1: unadjusted; Model 2: adjusted for age and sex; Model 3: adjusted for variables included in Model 2 and diabetes, hypercholesterolemia, stroke, hypoglycemic drugs, cholesterol-lowering drugs, body mass index, systolic blood pressure, diastolic blood pressure, white blood cell, hemoglobin, albumin, estimated glomerular filtration rate, fibrinogen, C-reactive protein, fasting plasma glucose and hemoglobin A1c for all-cause death; adjusted for age, hypertension, diabetes, stroke, hypoglycemic drugs, systolic blood pressure, diastolic blood pressure, white blood cell, hemoglobin, platelets, alanine transaminase, albumin, estimated glomerular filtration rate, fibrinogen and C-reactive protein for cardiovascular death. SUA, serum uric acid; HR, hazard ratio; CI, confidence interval.

### 3.3 Subgroup analysis and sensitivity analysis

As shown in **Table 3**. Subgroup analysis showed that the association between SUA and all-cause death risk was still statistically significant in individuals over 60 years old, male, with hypertension, without diabetes and with CKD (P < 0.05), while the association with cardiovascular death risk only persisted in individuals over 60 years old and male (P < 0.05). As shown in **Table 4**, further sensitivity analysis showed that SUA was still closely associated with all-cause and cardiovascular death, whether as a continuous variable or a classified variable (Model 3, as a continuous variable, HR: 1.002, 95% CI: 1.001-1.003, P = 0.001; HR: 1.002, 95% CI: 1.000-1.003, P = 0.015; as a classified variable, HR: 1.381, 95% CI: 1.091-1.748, P = 0.007; HR: 1.430, 95% CI: 1.010-2.025, P = 0.044).

**Table 3:** Subgroups analysis of association of SUA with all-cause and cardiovascular death.

	All-cause death					Cardiovascular death				
	Q1	Q2	Q3	Q4	P trend	Q1	Q2	Q3	Q4	P trend
	Ref.	HR (95% CI)	HR (95% CI)	HR (95% CI)		Ref.	HR (95% CI)	HR (95% CI)	HR (95% CI)	
Age										
≤ 60 years	1.0	0.969 (0.390, 2.407)	1.935 (0.766, 4.892)	2.353 (0.943, 5.874)	0.094	1.0	1.027 (0.267, 3.949)	1.069 (0.259, 4.421)	1.479 (0.361, 6.061)	0.931
> 60 years	1.0	0.818 (0.636, 1.052)	1.037 (0.809, 1.331)	1.355 (1.070, 1.715)*	0.001	1.0	0.705 (0.480, 1.034)	0.747 (0.500, 1.117)	1.426 (1.011, 2.011)*	< 0.001
Sex										
Male	1.0	0.822 (0.603, 1.119)	1.216 (0.909, 1.627)	1.537 (1.168, 2.022)**	< 0.001	1.0	0.701 (0.440, 1.119)	0.846 (0.532, 1.346)	1.521 (1.010, 2.291)*	0.001
Female	1.0	0.806 (0.527, 1.233)	0.663 (0.411, 1.069)	1.005 (0.624, 1.619)	0.270	1.0	0.610 (0.311, 1.195)	0.434 (0.195, 0.969)*	0.687 (0.324, 1.454)	0.199
Hypertension										
Yes	1.0	0.856 (0.636, 1.152)	1.014 (0.752, 1.367)	1.352 (1.013, 1.805)*	0.010	1.0	0.871 (0.553, 1.372)	0.944 (0.596, 1.494)	1.372 (0.893, 2.109)	0.119
No	1.0	0.757 (0.491, 1.167)	1.235 (0.808, 1.888)	1.343 (0.836, 2.160)	0.111	1.0	0.630 (0.314, 1.263)	0.361 (0.144, 0.903)*	1.128 (0.537, 2.369)	0.065
Diabetes										
Yes	1.0	0.719 (0.477, 1.082)	0.978 (0.630, 1.520)	1.417 (0.959, 2.094)	0.006	1.0	0.576 (0.300, 1.107)	0.560 (0.268, 1.172)	1.049 (0.576, 1.910)	0.108
No	1.0	0.785 (0.582, 1.059)	1.058 (0.793, 1.411)	1.353 (1.012, 1.807)*	0.004	1.0	0.886 (0.556, 1.411)	0.919 (0.571, 1.479)	1.482 (0.941, 2.334)	0.083
CKD										
Yes	1.0	1.307 (0.777, 2.199)	1.302 (0.770, 2.201)	1.752 (1.107, 2.770)*	0.069	1.0	0.754 (0.332, 1.712)	0.886 (0.404, 1.944)	1.312 (0.688, 2.502)	0.290
No	1.0	0.688 (0.521, 0.909)**	0.989 (0.750, 1.303)	1.009 (0.743, 1.372)	0.021	1.0	0.801 (0.524, 1.226)	0.805 (0.503, 1.288)	1.101 (0.678, 1.787)	0.473

The model used in the subgroups analysis consisted of all covariates used in model 3 in **Table 2** except for the variables that were used for stratification. SUA, serum uric acid; HR, hazard ratio; CI, confidence interval. *P < 0.05, **P < 0.01.

**Table 4:** Association between SUA with all-cause and cardiovascular death after excluding individuals with eGFR < 30 ml/min/1.73m^2^.

	Model 1	P value	Model 2	P value	Model 3	P value
	HR (95% CI)		HR (95% CI)		HR (95% CI)	
All-cause death						
Q1	Ref.	–	Ref.	–	Ref.	–
Q2	0.824 (0.648, 1.048)	0.115	0.773 (0.607, 0.983)	0.036	0.843 (0.658, 1.079)	0.175
Q3	1.085 (0.857, 1.373)	0.498	0.978 (0.773, 1.238)	0.854	1.126 (0.883, 1.436)	0.337
Q4	1.371 (1.094, 1.718)	0.006	1.294 (1.032, 1.622)	0.026	1.381 (1.091, 1.748)	0.007
P for trend	–	< 0.001	–	< 0.001	–	0.001
per 1 unit increment	1.002 (1.001, 1.003)	< 0.001	1.002 (1.001, 1.002)	0.002	1.002 (1.001, 1.003)	0.001
Cardiovascular death						
Q1	Ref.	–	Ref.	–	Ref.	–
Q2	0.831 (0.571, 1.211)	0.336	0.784 (0.538, 1.142)	0.204	0.737 (0.504, 1.078)	0.116
Q3	0.864 (0.584, 1.278)	0.464	0.778 (0.526, 1.152)	0.211	0.816 (0.550, 1.209)	0.310
Q4	1.510 (1.069, 2.134)	0.019	1.425 (1.008, 2.014)	0.045	1.430 (1.010, 2.025)	0.044
P for trend	–	0.004	–	0.003	–	0.002
per 1 unit increment	1.002 (1.001, 1.004)	0.003	1.002 (1.000, 1.003)	0.011	1.002 (1.000, 1.003)	0.015

Model 1: unadjusted; Model 2: adjusted for age and sex; Model 3: adjusted for variables included in Model 2 and diabetes, hypercholesterolemia, stroke, hypoglycemic drugs, cholesterol-lowering drugs, body mass index, systolic blood pressure, diastolic blood pressure, white blood cell, hemoglobin, albumin, estimated glomerular filtration rate, fibrinogen, C-reactive protein, fasting plasma glucose and hemoglobin A1c for all-cause death; adjusted for age, hypertension, diabetes, stroke, hypoglycemic drugs, systolic blood pressure, diastolic blood pressure, white blood cell, hemoglobin, platelets, alanine transaminase, albumin, eGFR, fibrinogen and C-reactive protein for cardiovascular death. SUA, serum uric acid; eGFR, estimated glomerular filtration rate; HR, hazard ratio; CI, confidence interval.

### 3.4 RCS and threshold effect analysis

**Figure 3:**
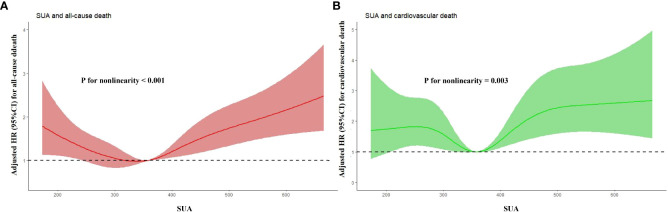
RCS analysis of association of SUA with all-cause **(A)** and cardiovascular death **(B)**. RCS, restricted cubic spline; SUA, serum uric acid; HR, hazard ratio; CI, confidence interval.

As shown in **Figure 3**. RCS analysis revealed that SUA had a nonlinear association with all-cause and cardiovascular death risk (P for nonlinearity < 0.01). As shown in **Table 5**, threshold effect analysis showed that SUA below 345 umol/L was negatively associated with all-cause and cardiovascular death risk (HR: 0.997, 95% CI: 0.995-0.999, P = 0.016; HR: 0.996, 95% CI: 0.993-1.000, P = 0.040), while SUA above 345 umol/L was positively associated with all-cause and cardiovascular death risk (HR: 1.003, 95% CI: 1.002-1.004, P < 0.001; HR: 1.003, 95% CI: 1.002-1.005, P < 0.001), and the 2-piecewise regression model was better than the 1-line regression model (P for likelihood ratio test < 0.05).

**Table 5:** Threshold effect analysis of SUA on all-cause and cardiovascular death.

	All-cause death		Cardiovascular death	
	HR (95% CI)	P value	HR (95% CI)	P value
1-line regression model	1.002 (1.001, 1.003)	0.001	1.002 (1.000, 1.003)	0.015
2-piecewise regression model				
Inflection point (K)	345.00		345.00	
< K segment	0.997 (0.995-0.999)	0.016	0.996 (0.993-1.000)	0.040
> K segment	1.003 (1.002-1.004)	< 0.001	1.003 (1.002-1.005)	< 0.001
P for likelihood ratio test		< 0.05		< 0.05

The analysis was adjusted for variables included in model 3 in **Table 2**. SUA, serum uric acid; HR, hazard ratio; CI, confidence interval.

## 4 Discussion

In this prospective cohort study based on population survey data, we not only found that the association between SUA and all-cause and cardiovascular death was statistically significant in the analysis of the total population and several subgroups, but also found that there was a U-shaped nonlinear association between SUA and the risk of all-cause and cardiovascular death, which deserved us to pay more attention to the influence of different levels of SUA on individuals with CHD.

Current studies have shown that SUA is closely related to many metabolic-related diseases. For example, Li et al. confirmed that a higher level of SUA had an exact causal relationship with a higher risk of gout and kidney calculi after a general review of the evidence from observational studies, randomized controlled trials and Mendelian randomized studies (14). In addition, Hong et al. also found in a Mendelian randomized study that the increased SUA level was closely related to the higher risk of atrial fibrillation (15). Besides, Weng et al. revealed a positive causal relationship between SUA and venous thromboembolism in a cohort and Mendelian randomized study (16). And the correlation between SUA and cardiovascular events, especially sudden cardiac death, was subsequently confirmed (17). However, the correlation between SUA and all-cause death and cardiovascular death has not been reached. Several studies have shown that SUA level is positively correlated with the risk of all-cause death or cardiovascular death. For example, after 985 peritoneal dialysis patients were followed up for a median of 25.3 months, Xia et al. found that the increase of baseline SUA level was an independent risk factor for all-cause death and cardiovascular death (18). In another small sample study, Feng et al. also found that a higher level of SUA was related to the increase of all-cause mortality in peritoneal dialysis patients (19). In addition, Wang et al. also reached a conclusion in a meta-analysis, that is, for every 1 mg/mL increase in SUA, the cardiovascular mortality and all-cause mortality increased by 12% and 20% respectively (8). Nevertheless, another study showed that higher levels of SUA were associated with lower mortality. For example, Latif et al. unexpectedly found that higher SUA levels were closely related to lower risk of all-cause death and cardiovascular death in a large prospective cohort study involving 4637 hemodialysis patients, so they thought that this unusual research result should be the subject of further research (12). Furthermore, higher levels of SUA in hemodialysis patients may represent stronger antioxidant capacity and better nutritional status, which can be confirmed by the conclusion that SUA is positively correlated with blood phosphorus levels, which also reflects the existence of SUA paradox in hemodialysis patients (12). Similar to this study, our study showed that SUA was not associated with the risk of cardiovascular death in patients with CKD, suggesting that high levels of SUA might have a cardiovascular protective effect in dialysis or CKD patients. However, low levels of SUA may increase the risk of cardiovascular death through low antioxidant capacity or low nutritional status. In addition, even some studies have not found the correlation between SUA and all-cause death or cardiovascular death (10, 11). At present, the conclusion accepted by most people is that there is a nonlinear U-shaped relationship between SUA and all-cause death and cardiovascular death. For example, in a follow-up cohort of 2060721.9 person-years, Cho et al. confirmed the U-shaped correlation between SUA level and death risk, that is, lower or higher SUA was closely related to higher death risk (20). Another cohort study also showed that both lower and higher SUA levels were associated with increased all-cause and cardiovascular mortality, which further supported the U-shaped correlation between SUA and mortality (21). However, the above studies did not explore the relationship between SUA and the risk of death in people with CHD, while our study just made up for this. In this study, we not only found the significant correlation between SUA and all-cause death and cardiovascular death of individuals with CHD, but also further found that these correlations existed stably in various subgroups, and even revealed the approximate U-shaped nonlinear correlation and threshold effect between SUA and all-cause and cardiovascular death, which laid the foundation for future multi-center studies. Moreover, because the circulation levels of SUA in patients with eGFR < 30ml/min/1.73m^2^ are very different from that of other people, severe renal dysfunction has a great impact on the levels of SUA. Therefore, in order to prevent the main results of the study from being affected by renal failure, we excluded patients with eGFR < 30ml/min/1.73m^2^ from sensitivity analysis to re-verify the correlation between SUA and all-cause and cardiovascular death risk in patients with CHD, and the results showed that higher levels of SUA were still closely associated with higher risk of all-cause and cardiovascular death, suggesting that this correlation might not be mediated by severe renal dysfunction. In addition, our study was similar to the conclusions of the above two studies, that is, we also found an approximate U-shaped nonlinear association between SUA and all-cause and cardiovascular death. However, Cho et al. did not further explore the threshold effects between SUA and all-cause and cardiovascular death (20). Although Hu et al. continued to explore this point, they conducted the analysis in the general population, and they found that the inflection point of threshold effect was 339 umo/L and that SUA was both positively associated with all-cause and cardiovascular death at higher than 339 umo/L, whereas it was negatively associated with all-cause death only at lower than 339 umo/L, which was not entirely consistent with our findings (21). In our study, we confirmed that the inflection point of threshold effect between SUA and all-cause and cardiovascular death in people with CHD was 345 umo/L, and that SUA lower than 345 umo/L was negatively correlated with all-cause and cardiovascular death, while SUA higher than 345 umo/L was positively correlated with all-cause and cardiovascular death.

Although we have confirmed the correlation between SUA and all-cause and cardiovascular death in people with CHD, the mechanism driving these associations is still unclear. For a high level of SUA, it can lead to the occurrence and progress of some metabolic related diseases, which are closely related to the risk of death, so we guessed that SUA could indirectly increase the risk of death through these diseases. In addition, several studies have shown that high levels of SUA can activate oxidative stress and inflammatory stress, such as activation and enhancement of interleukin-1 β, cyclooxygenase-2, reactive oxygen species and renin-angiotensin system, thus promoting endothelial dysfunction, which may eventually lead to an increase in the risk of death (22, 23). For the lower levels of SUA, some studies show that it can reflect the nutritional status and antioxidant capacity of individuals. The lower levels of SUA often represent malnutrition and weak antioxidant capacity, so these people may lack some vitamins at the same time (such as vitamin C and vitamin D), which may induce oxidative stress and endothelial function damage, and eventually lead to the occurrence of some metabolic diseases and increased risk of death (24–30). Nevertheless, we can’t say exactly that this is the potential mechanism, and more research is needed to further explore.

In this cohort study, we have gained some research results, but several shortcomings of this study were inevitable. Firstly, this was a study based on population survey data, which was somewhat different from the data of hospitalized patients with CHD, so the conclusion need to be verified in hospitalized patients with CHD before extrapolation. Secondly, because the baseline data came from population survey, although the data of blood markers were comprehensive, there was a lack of echocardiography and coronary angiography data. Thirdly, the causal association cannot be determined by observational research, so genetic association research is needed to further confirm their relationship. Fourthly, because in this study, we only excluded individuals who lacked baseline SUA data as well as those who were lost to follow-up, and these data did not include data on gout and drugs that might affect SUA levels, so we were not sure whether patients with gout or patients using drugs that affected SUA levels were included, but we speculated that there might be some gout patients among these participants, and there might also be some participants who were using drugs that affected SUA levels. However, it is not clear whether these conditions will affect the main results, so we will make up for these defects and further improve the research design in future research to make the results more generalizable.

## 5 Conclusions

In this prospective cohort study, we found that SUA had a U-shaped nonlinear association with all-cause and cardiovascular death risk in individuals with CHD, which indicated that too high or too low SUA had an impact on premature death and excessive mortality of individuals with CHD, which also provided a reference for formulating treatment strategies and precise treatment that matched different populations.

## Data availability statement

The original contributions presented in the study are included in the article/supplementary material. Further inquiries can be directed to the corresponding authors.

## Ethics statement

The studies involving humans were approved by the National Center for Health Statistics of the Center for Disease Control and Prevention Institutional Review Board. The patients/participants provided their written informed consent to participate in this study. The studies were conducted in accordance with the Declaration of Helsinki.

## Author contributions

XJY: Data curation, Writing - original draft, Writing - review & editing. JG: Data curation, Writing - original draft, Writing - review & editing. ZWW: Data curation, Writing - original draft, Writing - review & editing, Conceptualization, Methodology, Software, Formal analysis, Visualization. QYW: Writing - review & editing, Conceptualization, Validation, Funding acquisition, Project administration, Supervision. CJQ: Writing - review & editing, Conceptualization, Validation, Funding acquisition, Project administration, Supervision. FFW: Writing - review & editing, Conceptualization, Validation, Funding acquisition, Project administration, Supervision.
